# Ethnic/Racial Disparities in Pancreatic Cancer Mortality Across the United States: A National Inpatient Sample Database Analysis

**DOI:** 10.7759/cureus.77389

**Published:** 2025-01-13

**Authors:** Catherine D Fuko, Hezborn M Magacha, Gideon Noah, Obinna V Ikwuka

**Affiliations:** 1 Public Health (Biostatistics and Epidemiology), East Tennessee State University, Johnson City, USA; 2 College of Public Health, East Tennessee State University, Johnson City, USA; 3 Internal Medicine, East Tennessee State University, Quillen College of Medicine, Johnson City, USA

**Keywords:** ethnic disaprities, inpatient mortality, inpatient outcomes, nationwide inpatient sample (nis), pancreatic cancer mortality

## Abstract

Background and objective(s)

Pancreatic cancer remains a significant contributor to cancer-related mortality in the United States, with notable racial disparities in both incidence and mortality rates. This study aimed to investigate potential racial disparities in pancreatic cancer mortality across different racial/ethnic groups in the United States by using the National Inpatient Sample (NIS) database of 2016-2020.

Methods

Using data from the National Inpatient Sample (NIS) database, we identified 32,357 individuals aged 18 years and older with a primary diagnosis of pancreatic cancer between 2016 and 2020. The sample was weighted to be nationally representative. Multivariate logistic regression analyses were performed to examine the association between race/ethnicity and pancreatic cancer mortality, adjusting for potential confounding factors such as age, gender, and patient location.

Results

Among the study population, 72.11% were White individuals, 14.48% were Black/African American individuals, 9.38% were Hispanic individuals, 3.67% were Asian/Pacific Islander individuals, and 0.36% were Native American individuals. Black/African American individuals demonstrated significantly higher odds of mortality compared to White individuals (adjusted odds ratio (OR)=1.339, 95% CI: 1.324-1.478, p < 0.0001). Asian/Pacific Islander individuals also exhibited higher odds of mortality (adjusted (OR)=1.442, 95% CI: 1.308-1.590, p < 0.0001). No statistically significant differences were observed for Hispanic or Native American individuals compared to White individuals.

Conclusions

This study provided evidence of racial disparities in pancreatic cancer mortality, with Black/African American and Asian/Pacific Islander individuals facing significantly higher odds of mortality compared to White individuals. These findings underscored the need for targeted interventions and policies to address these inequities and promote health equity in pancreatic cancer outcomes.

## Introduction

Pancreatic cancer ranks as the third highest contributor to cancer-related mortalities in the United States, affecting both males and females [1]. It continues to pose a significant threat among gastrointestinal (GI) cancers. There remains a lack of understanding regarding racial disparities in both its incidence and mortality rates [2]. According to the National Cancer Institute (NIH) under the Surveillance, Epidemiology, and End Results program (SEER), the incidence of new pancreatic cancer cases stood at 13.3 per 100,000 individuals annually, encompassing both men and women among all races/ethnicities. Similarly, the mortality rate was recorded at 11.1 per 100,000 individuals annually. These figures are standardized for age and derived from cases and fatalities between 2016 and 2020. In the year 2020, it's estimated that there were approximately 95,389 individuals residing in the United States who had been diagnosed with pancreatic cancer. Studies show that around 1.7 percent of individuals, irrespective of gender, are projected to receive a pancreatic cancer diagnosis at some stage in their lives, as per data spanning from 2017 to 2019 [3]. 

Factors contributing to pancreatic cancer risk encompass genetic and non-genetic elements. Genetic syndromes, such as those resulting from inherited mutations in genes like STK11, BRCA1, BRCA2, PALB2, CDKN2A, and DNA repair genes, notably elevate the risk of pancreatic cancer, particularly in individuals with a family history of pancreatic cancer among first-degree relatives. Data regarding variations in pancreatic cancer risk among different racial or ethnic groups within these high-risk syndromes are currently unavailable [4-7]. The non-genetic risk factors for pancreatic cancer include smoking (tobacco use), obesity, chronic pancreatitis, alcohol abuse, and diabetes [7,8]. Research has demonstrated that smoking is one-quarter responsible for pancreatic cancer and can raise the risk of the disease. Epidemiological data indicates that individuals who abuse alcohol have a higher incidence and mortality rate of pancreatic cancer compared to non-drinkers. This association is partly attributed to chronic pancreatitis, which is known to be a risk factor for pancreatic cancer and is often linked with heavy alcohol consumption [9].

CDC defines health disparities as avoidable variations that socially disadvantaged communities face in terms of the burden of illness, injury, violence, or opportunities to reach optimal health [10]. The impact of cancer varies significantly, with social structures and practices exerting influence on preventive measures, access to care, treatment options, and ultimately, survival rates across different cancer types. Factors such as cancer stage, health-related behaviors, presence of comorbidities, and treatment decisions emerge as key determinants in understanding socioeconomic and sociodemographic disparities in cancer outcomes [11,12]. Black/African American people have greater incidence rates of pancreatic cancer than other racial and ethnic groups, according to research on racial/ethnic differences in the disease. Also, Black/African American individuals have higher death rates from pancreatic cancer [13,14]. The age-adjusted pancreatic cancer mortality rate reveals a disparity, with Black individuals experiencing a rate of 13.3 deaths per 100,000 people, which is higher than the rate of 11.0 per 100,000 seen in White individuals [15]. Data from the National Cancer Institute's SEER program indicates that Black/African American individuals exhibit the highest incidence rate of pancreatic cancer among all ethnic and racial groups in the United States [16,17]. Black/African American individuals have a 30-70% higher incidence risk of pancreatic cancer than other racial groups in the US, according to the SEER 21 database [16,18]. They face the worst pancreatic cancer prognosis compared to all other population groups, characterized by a higher prevalence of advanced stage and unresectable disease upon presentation, along with less access to surgical interventions. Treatment gaps across various racial and socioeconomic populations could be one cause for these survival differences [15,19].

Research reveals that Black/African American individuals are less likely to undergo pancreatic resection in comparison to Caucasian individuals, a factor that can significantly influence the outcome [20]. Factors like smoking, obesity, and tumor features are some of the other explanations for the variations in survival between racial and ethnic groups [14]. The relationships between racial disparities and pancreatic cancer incidence and mortality rates are currently understood to be complex, with multiple variables interacting with one another. Socioeconomic factors play a significant role in contributing to racial disparities in pancreatic cancer outcomes. Black/African American individuals are disproportionately affected by poverty, lack of health insurance (uninsured and underinsured), limited access to healthcare facilities, and disparities in the quality of care. These socioeconomic disadvantages often result in delays in diagnosis, suboptimal treatment utilization, and poorer overall survival outcomes among Black/African American patients with pancreatic cancer. Unlike breast and colon cancer, for which screening methods can identify early-stage disease, there is currently no screening modality available for pancreatic cancer. Consequently, any disparities in outcomes associated with pancreatic cancer cannot be attributed to a deficiency in screening efforts [8,21].

The cancer stage at diagnosis will determine treatment options and survival rates. According to SEER, the relative survival rate of pancreatic cancer is 12.5%. The earlier pancreatic cancer is detected, the greater the likelihood of a person surviving beyond five years following diagnosis. Around 12.9% of pancreatic cancer cases are diagnosed at the local stage, which is a small percentage; hence it highlights the challenges linked to early detection. The five-year relative survival rate for localized/in situ pancreatic cancer stands at 44.3% [3].

Despite progress in comprehending the disease, there is still a notable absence of comprehensive studies that investigate the unequal impact of pancreatic cancer among various demographic groups [2]. The racial disparities in pancreatic cancer mortality involve investigating and understanding which racial and ethnic groups have high pancreatic cancer mortality rates and why certain racial and ethnic groups, particularly Black/African American individuals, experience higher mortality rates of pancreatic cancer compared to other populations. These disparities may encompass variations in incidence rates, timely diagnosis and treatment access, and survival outcomes. Addressing these gaps is crucial for developing targeted interventions and policies aimed at reducing the disproportionate impact of pancreatic cancer on marginalized populations and ultimately working towards achieving health equity in cancer prevention and outcomes.

This study hypothesized that the mortality rates of pancreatic cancer differ significantly by race in the US, with Black/African American people having greater rates than White people and members of other ethnic groups. The objective of this study was to determine mortality rates of pancreatic cancer across different racial and ethnic groups.

## Materials and methods

The data was obtained from The National (Nationwide) Inpatient Sample (NIS), which is a nationally representative sample of hospital inpatient stays in the United States. The NIS is one of the databases created by the Healthcare Cost and Utilization Project (HCUP), and it is the largest inpatient database available. The NIS database spans from the year 1988 to 2020, with an increasing number of participating states over the years, starting from eight states to now include 48 states plus the District of Columbia. NIS approximates a 20-percent stratified sample of discharges from community hospitals in the United States, with rehabilitation and long-term acute care hospitals excluded. By using the NIS data, we aimed to shed light on potential disparities in pancreatic cancer mortality among different racial groups.

A pancreatic cancer diagnosis was identified using the International Classification of Diseases (ICD-10), 10th version, World Health Organization (WHO) as the primary diagnostic for hospital admissions in healthcare institutions across the United States from the years 2016-2020 [22]. The final sample included 32,357 individuals aged 18 years and older with a primary diagnosis of pancreatic cancer. To ensure the study sample was representative of the target population, the sample was weighted using a weighting variable, resulting in a sample size of 161,785 individuals.

The dependent/outcome variable was 'died,' categorized as either 'died' or 'did not die.' The primary independent variable was race, classified as 1=White individuals, 2=Black individuals, 3=Hispanic individuals, 4=Asian or Pacific Islander individuals, and 5=Native American individuals. Additional variables included age, which was grouped into the following ranges: 1=18-29, 2=30-39, 3=40-49, 4=50-59, 5=60-69, and 7=70+. Gender was categorized as 0=male and 1=female, and the patient's location was recorded as 1=urban and 0=rural. The variable 'year' encompassed the years from 2016 to 2020.

Using SAS statistical software version 9.4 (SAS Inc., Cary, NC), all analyses were conducted. The tests were performed at 95% confidence intervals (CI) with a 5% significance level. A descriptive analysis was conducted to show the distribution of the variables in the study population. The chi-square test was used to reveal if race and mortality were significantly related. Bivariate logistic regression was employed to examine the association between race/ethnicity and pancreatic cancer mortality, alongside other variables such as age, gender, and patient location. The multivariate (adjusted) analysis then incorporated these various patient characteristics as covariates in the logistic regression model to isolate the independent effects of each factor on the probability of dying due to pancreatic cancer. Controlling these demographics and patient residential locations provided estimates of the relationship between race/ethnicity and pancreatic cancer mortality independent of these other influences by using odds ratios and 95% confidence intervals.

## Results

Descriptive analysis

Table 1 shows the distribution of variables, a descriptive analysis of the study population. The descriptive statistics of the study population for pancreatic cancer reveal an almost equal gender distribution, with males comprising 50.5% (n=16341) and females 49.5% (n=16016). The age distribution demonstrated that the age group 70+ (n=15259, 47.16%) represents the largest portion of the population with pancreatic cancer, followed by the 60-69 age range (n=10075, 31.14%). Age group 50-59 (n=5257, 16.25%) makes up a smaller but notable proportion of the population; meanwhile, the younger age groups 40-49 (n=1423, 4.40%), 30-39 age range (n=259, 0.80%), and 18-29 age range (n=84, 0.26%) have progressively fewer cases. The age distribution skews heavily toward older patients, with over 78% of the sample being 60 years of age or older. Looking at race, most individuals were White individuals (n=23333, 72.11%), followed by Black/African American individuals (n=4686, 14.48%), Hispanic individuals (n=3035, 9.38%), Asian/Pacific Islander individuals (n=1187, 3.67%), and Native American individuals (n=116, 0.36%). About 6.14% (n = 1987) of hospitalized individuals died during the hospital stay.

**Table 1:** Descriptive analysis of pancreatic cancer cases, National Inpatient Sample, 2016–2020.

Variable	Value	Unweighted frequency	Unweighted %	Weighted frequency	Weighted %
Gender	Male	16341	50.50	81705	50.50
	Female	16016	49.50	80080	49.50
Age	18-29	84	0.26	420	0.26
	30-39	259	0.80	1295	0.80
	40-49	1423	4.40	7115	4.40
	50-59	5257	16.25	26285	16.25
	60-69	10075	31.14	50375	31.14
	70+	15259	47.16	76295	47.16
Race/Ethnicity	White individuals	23333	72.11	116665	72.11
	Black individuals	4686	14.48	23430	14.48
	Hispanic individuals	3035	9.38	15175	9.38
	Asian/Pacific Islander individuals	1187	3.67	5935	3.67
	Native American individuals	116	0.36	580	0.36
Died	Yes	1987	6.14	9935	6.14
	No	30370	93.86	151850	93.86
Year	2016	6304	19.48	31520	19.48
	2017	6482	20.03	32410	20.03
	2018	6496	20.08	32480	20.08
	2019	6701	20.71	33505	20.71
	2020	6374	19.70	31870	19.70
Patient location	Rural	2966	9.17	14830	9.17
	Urban	29391	90.83	146955	90.83

**Figure 1:**
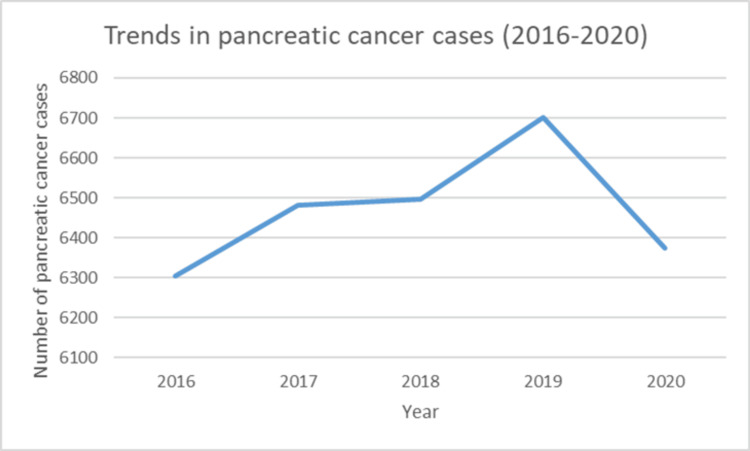
Trends in pancreatic cancer cases, National Inpatient Sample, 2016-2020.

The sample size is distributed relatively evenly across the years 2016 to 2020. In 2016, there were 6,304 (19.48%) individuals. The following year, 2017, there was a slight increase with 6,482 individuals (20.03%). This upward trend continued in 2018 with 6,496 individuals (20.08%). The peak was reached in 2019, which recorded 6,701 individuals (20.71%). Finally, in 2020 there was a decrease to 6,374 cases (19.70%). Figure 1 (trend chart) demonstrates a visual representation of these trends. The data on patient location shows a clear distinction between rural and urban settings. Around 90.83% (n=29,391) were from urban areas, and 9.17% (n=2,966) were from rural areas.

Bivariate analysis

Black/African American individuals demonstrated significantly higher odds of mortality compared to the reference group (White individuals), with an increase in odds of 30.9%. The odds ratio (OR=1.309, 95% CI: 1.240, 1.383, p=<0.0001). Also, Asian/Pacific Islander individuals demonstrated an increase in odds of 39.1% compared to White individuals (OR=1.391, 95% CI=1.262 to 1.534, and p=<0.0001). Hispanic individuals did not show a significant difference in odds compared to the reference group (White individuals), OR=0.989, 95% CI=0.920, 1.064, p-value=0.7728). Also, the Native American individuals did not exhibit significant statistics. The OR=1.198, with a 95% CI=0.868, 1.653, p-value=0.2722. Other variables that showed statistical significance included age and gender (Table 2).

**Table 2:** Bivariate and multivariate analysis for odds ratio, confidence intervals, and p-values of pancreatic cancer cases, National Inpatient Sample, 2016-2020.

Unadjusted			Adjusted			
	Point estimate (odds ratio)	Estimate confidence interval	p-value	Point estimate (odds ratio)	Estimate confidence interval	p-value
Race/Ethnicity: White individuals (reference)						
Black/African American individuals	1.309	1.240, 1.383	< .0001>	1.339	1.324 1.478	< .0001>
Hispanic individuals	0.989	0.920, 1.064	0.7728	1.056	0.982 1.136	0.1433
Asian/Pacific Islander individuals	1.391	1.262, 1.534	< .0001>	1.442	1.308 1.590	< .0001>
Native American individuals	1.198	0.868, 1.653	0.2722	1.255	0.908 1.733	0.1689
Age (years): 70+ (reference)						
18-29	0.513	0.306, 0.859	0.0112	0.520	0.310, 0.872	0.0132
30-39	0.441	0.322, 0.605	< .0001>	0.416	0.303, 0.571	< .0001>
40-49	0.663	0.591, 0.743	< .0001>	0.622	0.554, 0.698	< .0001>
50-59	0.805	0.758, 0.855	< .0001>	0.759	0.715, 0.807	< .0001>
60-69	0.867	0.828, 0.909	< .0001>	0.835	0.797, 0.875	< .0001>
Gender: Male (reference)						
Female	0.771	0.740, 0.803	< .0001>	0.748	0.718, 0.779	< .0001>
Patient location: Urban (reference)						
Rural	1.071	1.000, 1.147	0.0513	1.337	1.283, 1.394	0.0067

Multivariate analysis

In the adjusted analysis, Black/African American individuals demonstrated 33.9% higher odds of dying compared to White individuals (OR=1.339, 95% CI: 1.324, 1.478, p=<0.0001). Asian/Pacific Islander individuals demonstrated an increase in odds of 44.2% of dying compared to White individuals (OR=1.442, 95% CI: 1.308, 1.590, p=<0.0001). Hispanic individuals did not show a significant difference in odds compared to White individuals (OR=1.056, 95% CI: 0.982, 1.136, p-value=0.1433). Native American individuals did not show a significant difference (OR=1.255, 95% CI: 0.908, 1.733, p-value=0.1689).

Younger age groups showed significantly lower odds of dying compared to the 70+ reference group. The 18-29 age group had an OR of 0.520 (95% CI: 0.310, 0.872, p=0.0132), a 48% decrease in odds. The 30-39 age group had an OR of 0.416 (95% CI: 0.303, 0.571, p<0.0001), indicating a 58.4% decrease in odds. The 40-49 age group had an OR of 0.622 (95% CI: 0.554, 0.698, p<0.0001), a 37.8% decrease in odds. The 50-59 age group had an OR of 0.759 (95% CI: 0.715, 0.807, p<0.0001), a 24.1% decrease in odds. The 60-69 age group had an OR of 0.835 (95% CI: 0.797, 0.875, p<0.0001), a 16.5% decrease in odds. This demonstrates a consistent trend of lower odds across age groups compared to the 70+ reference group. Females demonstrated a 25.2% decrease in odds of dying (OR=0.748, 95% CI: 0.718, 0.779, p<0.0001). Individuals from rural areas had significantly higher odds of dying compared to those from urban areas. A 33.7% increase in the odds of dying from pancreatic cancer (OR=1.337, 95% CI: 1.283, 1.394, p=0.0067) (Table 2).

## Discussion

The study explored the disparities in pancreatic cancer mortality across different racial groups in the United States, shedding light on an important yet understudied aspect of cancer epidemiology. The findings from this study provided important insights into the racial disparities that exist in pancreatic cancer mortality rates across different ethnic groups in the United States. The results demonstrated that Black/African American individuals face significantly higher odds of mortality from pancreatic cancer compared to White individuals, even after adjusting for other factors such as age, gender, and patient location. These findings contributed to the existing literature by reaffirming the significant impact of race on pancreatic cancer outcomes, particularly highlighting the disproportionately higher mortality rates among Black/African American individuals compared to White individuals and other ethnic groups. Several potential contributors may explain this disparity, including differences in tumor biology, differential access to high-quality care and treatment, and a higher prevalence of risk factors such as smoking and obesity [23-26].

Interestingly, Asian/Pacific Islander individuals also exhibited increased odds of pancreatic cancer mortality compared to White individuals in both the unadjusted and adjusted analyses. This finding warrants further investigation to understand the underlying factors driving this elevated risk. Potential explanations could include genetic or environmental factors specific to this racial group. In contrast, no significant differences in mortality odds were found between Hispanic individuals and White individuals after adjusting for demographic factors. Similarly, the sample size for Native American individuals was too small to detect any meaningful differences. Larger studies focused specifically on these populations are needed to fully explain potential disparities [25-27].

These findings underscored the pressing need to address the root causes of racial disparities in pancreatic cancer outcomes. Exploring the intersectionality of socioeconomic status, access to healthcare, and quality of care alongside race could provide valuable insights into the mechanisms driving differential outcomes in pancreatic cancer mortality. Moving forward, more efforts should focus on improving access to high-quality screening, early detection, and treatment for vulnerable populations. Additionally, targeted interventions aimed at modifying risk factors and raising awareness about pancreatic cancer symptoms may help mitigate these disparities.

One of the notable strengths of this study was the utilization of a nationally representative dataset, the National (Nationwide) Inpatient Sample (NIS), spanning from 1988 to 2020, of which this specific study analyzed the data from 2016 to 2020. By leveraging a large sample size and rigorous statistical analysis, the study offered strong evidence to support its findings. The inclusion of multiple variables such as race, age, gender, and patient location allows for a comprehensive examination of pancreatic cancer mortality disparities and their covariates. In addition to that, the study's findings were consistent with existing literature that has documented racial disparities in pancreatic cancer incidence and mortality, particularly the disproportionate burden among Black/African American populations [15,24-27].

Ethnic and racial disparities in pancreatic cancer mortality are significant across the United States, with evidence indicating that African American individuals experience notably higher mortality rates compared to other racial groups. According to the American Cancer Society, African American men and women have approximately 50% higher pancreatic cancer mortality rates than their White counterparts [28]. This disparity is attributed to a combination of factors, including differences in access to healthcare, socioeconomic status, and variations in the prevalence of risk factors such as diabetes and obesity [29]. Moreover, studies suggest that delays in diagnosis and treatment, compounded by structural inequities within the healthcare system, further exacerbate these disparities [27-30].

Potential limitations of this study include the use of administrative data, which may be subject to coding errors or incomplete information. Furthermore, the study did not account for factors such as socioeconomic status, health behaviors, or comorbidities, which could influence mortality rates. Future research should aim to incorporate these variables to gain a more comprehensive understanding of the complex interplay between race, social determinants of health, and pancreatic cancer outcomes.

Another limitation of this study was the lack of disaggregated data on specific racial/ethnic subgroups within broader categories such as "Asian/Pacific Islander" or "Hispanic" individuals. These broad categorizations may obscure potential heterogeneity and variations in pancreatic cancer mortality rates among different subpopulations within each category. For instance, the "Asian/Pacific Islander" group encompasses a diverse range of ethnicities, including East Asian, Southeast Asian, and Pacific Islander communities, which may have distinct risk profiles and cultural factors influencing pancreatic cancer outcomes. Similarly, the "Hispanic" category encompasses individuals from various national origins and backgrounds, each with unique sociocultural and environmental influences. By aggregating these subgroups into larger categories, the study may fail to capture potential nuances and variations in pancreatic cancer disparities, limiting the ability to tailor interventions and address the specific needs of individual subpopulations effectively.

The findings of this study had significant implications for public health policies and interventions aimed at addressing racial disparities in pancreatic cancer outcomes. By highlighting the disproportionate burden of pancreatic cancer mortality among Black/African American and Asian/Pacific Islander populations, this study underscored the need for targeted prevention, early detection, and treatment strategies tailored to these high-risk groups. Stakeholders, such as public health organizations, healthcare providers, and policymakers, can utilize this information to develop culturally appropriate educational campaigns and outreach programs to increase awareness about pancreatic cancer risk factors and the importance of early screening and diagnosis. Additionally, efforts should be made to address barriers to healthcare access and ensure equitable distribution of high-quality cancer care services, particularly in underserved communities.

## Conclusions

In conclusion, the persistent ethnic and racial disparities in pancreatic cancer mortality across the United States underscore a critical need for targeted interventions and policy changes. The higher mortality rates observed among African American individuals, coupled with the intricate interplay of socioeconomic, healthcare access, and biological factors, highlight significant inequities within the current healthcare system. Addressing these disparities requires a multifaceted approach, including improving access to timely and effective screening, enhancing patient education, and addressing systemic barriers to care. By focusing on these areas, it is possible to reduce the gap in pancreatic cancer outcomes and work toward more equitable healthcare for all racial and ethnic groups. Comprehensive strategies that encompass both prevention and treatment, alongside robust support systems, are essential to achieving meaningful progress in overcoming these disparities and ultimately improving survival rates for all affected populations.
